# From Across the Seas

**Published:** 1916-04-29

**Authors:** 


### FROM ACROSS THE SEAS.

### Where Babies Thrive—Antipodean Salaries—The Surgeon's Pledge.

### An American Baby Town.

In connection with the recent observance of Baby Week in Montclair, N.J., announcement is made that during 1915 the death rate of infants under one year of age in Montclair was 65 per 1,000 births, as compared with 123 per 1,000 for the State of New Jersey, 124 for the United States, 160 for Japan, 192 for Germany, and 332 for Chile. The deaths from diarrhœal disease of children under two years of age in Montclair decreased from 131 per 100,000 of population twenty-five years ago to twelve in 1915; while the number of deaths of children under five years of age decreased from 500 per 100,000 of population twenty-five years ago to 163 in 1915.—*From the "Medical Record," New York.*

### Antipodean Medical Salaries.

The salaries of resident medical hospital men in the home country differ widely at the present time, and are governed greatly by the standing of the institution making the appointment; the result being that a small and medically uninteresting hospital has to offer two or three times the amount of remuneration profferred by a more important and professionally influential institution. Judging from a series of advertisements appearing in the *Medical Journal of Australia*, Sydney, salaries offered are fairly uniform and range higher than would those of corresponding posts at home in normal times, but apparently the residents, though provided with lodging, are not always allowed board. For instance, the Broken Hill and District Hospital advertises for two junior resident medical officers, and offers £150 to £200 per annum "according to experience." Ladies will be accepted, also young graduates wishing to gain hospital experience before enlisting. Evidently a dearth of applications is expected.

The Hobart General Hospital requires a junior house surgeon, and the successful applicant is expected to stay for at least one year. As an encouragement a progressive salary is offered: £200 for first year, £225 for second, and £250 for third year, with furnished quarters (suitable for a single man), fuel, light, and water. Another appointment, presumably non-resident, is that of medical officer to the Texas District Hospital, Queensland, where the remuneration is £200 per annum, and instruments and drugs are provided by the hospital. Private practice, estimated at £600 per annum at lowest, is allowed, and the applicants are informed that it is a thriving district with no opposition, the nearest doctor being thirty miles distant.

### A Pledge for Surgeons.

The following fellowship pledge was adopted by the American College of Surgeons at its fourth Convocation recently held in Boston:

> Recognising that the American College of Surgeons seeks to develop, exemplify and enforce the highest traditions of our calling, I hereby pledge myself, as a condition of fellowship in the college, to live in strict accordance with all its principles, declarations, and regulations. In particular I pledge myself to pursue the practice of surgery with thorough self-restraint and to place the welfare of my patients above all else; to render willing help to my colleagues and to give freely my services to the needy. Moreover, I pledge myself to shun unwarranted publicity, dishonest money-seeking, and commercialism as disgraceful to our profession; to refuse utterly all secret money trades with consultants and practitioners; to make my fees commensurate with the services rendered and with the patient's rights, and to avoid discrediting my associates by taking unwarranted compensation.

The *Trained Nurse and Hospital Review*, New York, which reproduces the pledge, in dealing with the question of seemingly unnecessary operations, says: "We lived through the craze for removing ovaries some years ago. Lately the tonsils have been attacked and removed for such a variety of ailments that many protesting voices arose here and there in an effort to check the practice to some extent. If the surgeon's pledge will help to prevent unnecessary operations, those who planned it will deserve the thanks of thousands."

